# ULTIMATE-SHF trial (UdenafiL Therapy to Improve symptoMAtology, exercise Tolerance and hEmodynamics in patients with chronic systolic heart failure): study protocol for a randomized, placebo-controlled, double-blind trial

**DOI:** 10.1186/1745-6215-14-188

**Published:** 2013-06-22

**Authors:** Kyung-Hee Kim, Hyung-Kwan Kim, In-Chang Hwang, Seung-Pyo Lee, Hyun-Jai Cho, Hyun-Jae Kang, Yong-Jin Kim, Dae-Won Sohn

**Affiliations:** 1 Department of Internal Medicine, Seoul National University College of Medicine, Seoul 110-744, Korea; 2 Cardiovascular Center, Seoul National University Hospital, Seoul 110-744, Korea

**Keywords:** Phosphodiesterase type 5 inhibitor, Udenafil, Chronic heart failure, Exercise capacity

## Abstract

**Background:**

Over the last few years, the use of phosphodiesterase type 5 (PDE5) inhibitors has been expanded to management of various cardiovascular disorders beyond pulmonary arterial hypertension. This study is designed to investigate the ability of udenafil, a newly developed long-acting PDE5 inhibitor, to improve functional capacity and hemodynamic status in a cohort of chronic systolic heart failure (SHF) patients.

**Methods/design:**

Stable, chronic SHF patients will be randomly assigned to placebo (26 patients) or udenafil at a dose of 50 mg twice per day (26 patients) for the first 4 weeks followed by 100 mg twice daily for the next 8 weeks. Eligibility criteria will be age ≥18 years, clinical diagnosis of chronic SHF with current New York Heart Association class II to IV symptoms, left ventricular ejection fraction ≤ 40%, and experience of at least one of following during the 12 months prior to study entry: hospitalization for decompensated heart failure, acute treatment with intravenous loop diuretics or hemofiltration, or pulmonary artery systolic pressure ≥40mmHg on transthoracic echocardiography. Pharmacological therapy for SHF will be optimized in all patients at least 30 days before study entry. The primary outcome will be the change of maximal oxygen uptake, assessed by cardiopulmonary exercise testing. Secondary outcomes will include changes in ventilatory efficiency (minute ventilation/carbon dioxide production slope), left ventricular systolic and diastolic parameters, pulmonary artery systolic pressure, plasma concentration of brain natriuretic peptide, occurrence of mortality or hospitalization for heart failure, and the occurrence of any adverse event.

**Clinical trial registration:**

Unique identifier: NCT01646515

## Background

Heart failure is a leading cause of death that continues to cause a significant socioeconomic burden worldwide [1]. The prevalence of chronic heart failure (HF) in relation to left ventricular (LV) systolic dysfunction is estimated to be as high as 2 to 11% [2]. Despite the aggressive use of medications, such as angiotensin-converting enzyme inhibitors, β-blockers and spironolactone, proven to improve the survival of chronic HF patients with a reduced LV ejection fraction, mortality and morbidity remain high, and one of the potential reasons for this might be the development of pulmonary hypertension. According to a number of earlier works, up to 60% of patients with severe LV systolic dysfunction develop pulmonary hypertension [3,4]. Hence, it is conceivable that the development of pulmonary venous or mixed pulmonary venous/arterial hypertension, which results in HF symptoms such as dyspnea and limitation of exercise capacity, and consequently adversely affects quality of life and prognosis, is an important milestone in the progression of uncomplicated LV systolic dysfunction to clinically manifested HF. In this respect, development of a new therapeutic option that deals with the issue of pulmonary hypertension in association with LV systolic failure is both attractive and clinically relevant.

Phosphodiesterase type 5 (PDE5) is a key enzyme in the catabolism of cyclic guanine monophosphate (cGMP) and is predominantly abundant in the vascular smooth muscle cells of the pulmonary vasculature [5]. Given the important role of cGMP in the regulation of nitric oxide and that defective nitric oxide release is a major factor of vasoconstriction in chronic HF [6], the addition of PDE5 inhibitors to established medications for chronic HF could be a theoretically appealing treatment strategy. PDE5 inhibitors were initially introduced as a treatment option for erectile dysfunction, and even in the late 20th century this type of drug was believed to have little to contribute in the cardiology field. However, since PDE5 inhibitors were found to have beneficial effects on pulmonary arterial hypertension in man [7], and the confirmation of this finding in clinical trials [8,9], PDE5 inhibition is now considered a viable therapeutic option for the treatment of pulmonary arterial hypertension. In addition to their favorable therapeutic impacts on pulmonary arterial hypertension, PDE5 inhibitors have recently been shown to be effective therapeutics in patients with chronic systolic heart failure (SHF) [8,10].

**Figure 1:**
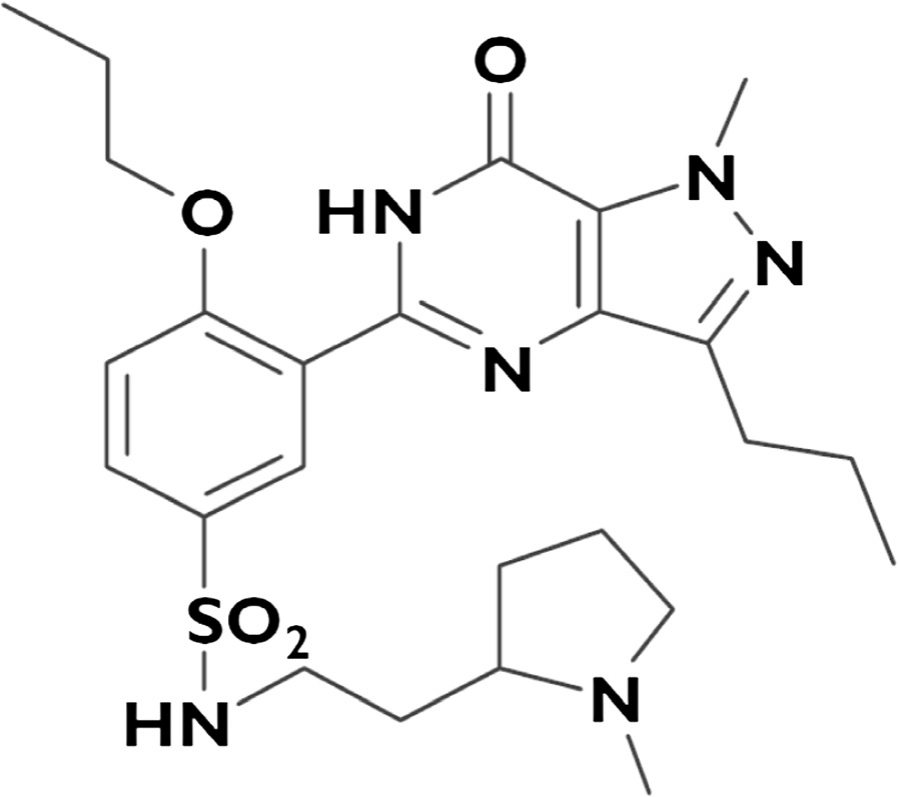
Chemical structure of udenafil.

Udenafil (Zydena®; Dong-A Pharmaceutical Company, Seoul, South Korea) is a newly developed long-acting PDE5 inhibitor [11,12], with efficacy and safety profiles comparable with those of other PDE5 inhibitors [13]. Udenafil is similar to sildenafil in molecular structures (Figure 1), and is comparable with sildenafil in terms of PDE5 selectivity and its broad range of safety margin [14]. Furthermore, laboratory data showed that udenafil inhibits ventricular hypertrophy and fibrosis in a rat HF model [15]. In the preclinical study, udenafil increased serum cGMP levels, decreased intracellular Ca^2+^ concentrations, and produced vasodilatation. In this proposed double-blind, randomized, placebo-controlled trial we hypothesize that udenafil will ameliorate symptoms, and improve exercise capacity and hemodynamic status in patients with chronic SHF (ULTIMATE-SHF trial; NCT01646515).

## Methods/design

### Study population

The ULTIMATE-SHF trial is designed as a prospective, single-center, randomized, double-blind, placebo-controlled study targeting patients with chronic SHF, in whom optimal medical therapy has been established. Patients with stable chronic SHF that meet the prespecified eligibility criteria and provide written informed consent will be enrolled (Table 1). Detailed information on benefits and risks will be provided to study candidates. Pharmacological therapy for heart failure will be optimized in all patients at least 30 days before study entry.

**Table 1:** Eligibility criteria

**Inclusion criteria**	**Exclusion criteria**
1. Able to provide written informed consent	1. FEV1 <80% of predicted value or FEV1/FVC <70% on spirometry at screening
2. Men and women ≥18 years old	2. Other valve diseases greater than mild stenosis and/or regurgitation
3. Established clinical diagnosis of chronic systolic heart failure with NYHA class II to IV symptoms currently	3. Neuromuscular, orthopedic, or other noncardiac conditions that prevent completion of a maximal exercise testing
4. Left ventricular ejection fraction ≤40%, as determined by echocardiography at the baseline echocardiographic examination	4. Infiltrative/ inflammatory myocardial or pericardial disease
	5. Primary pulmonary arteriopathy
5. Have experienced at least one of the following criteria in the 12 months before study entry:	6. Current use of nitrate preparation therapy (will be deleted) or other PDE5 inhibitors (that is, sildenafil, vardenafil, tadalafil) or cytochrome P450 3A4 inhibitors (ketoconazole, itraconazole, erythromycin)
- Hospitalization for decompensated heart failure	
- Acute treatment with intravenous loop diuretics or hemofiltration	7. Severe hypotension (SBP <90 mmHg or DBP <50 mmHg) or uncontrolled hypertension (SBP >180 mmHg or DBP >100 mmHg)
- Pulmonary artery systolic pressure ≥40 mmHg by echocardiography	8. Noncardiac illness with estimated life expectancy <1 year at the time of study entry, based on the judgment of the attending physician
6. Stable medical therapy for 1 month before study entry – no addition or removal or change in major class of medication dosage; that is, renin–angiotensin–aldosterone inhibitors, β-blockers	9. Known severe renal dysfunction (GFR <30 ml/minute/1.73 m^2^ by MDRD equation)
	10. Known severe liver disease (ALT or AST level >3 times the upper normal limit, alkaline phosphatase or total bilirubin >2 times the upper normal limit)
	11. Actively involved in any physical training program for at least 6 months before study entry
	12. Listed for heart transplantation

*ALT*, alanine aminotransferase; *AST*, aspartate aminotransferase; *DBP*, diastolic blood pressure; *FEV1*, forced expiratory volume in 1 second; *FVC*, forced vital capacity; *GFR*, glomerular filtration rate; *MDRD*, Modification of Diet in Renal Disease; *NYHA*, New York Heart Association; *SBP*, systolic blood pressure.

### Study design and medications

**Figure 2:**
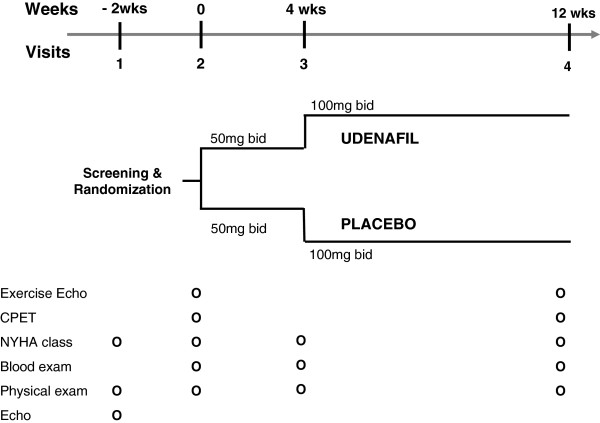
**Study visit schedule of the ULTIMATE-SHF trial.** bid, twice daily; CPET, cardiopulmonary exercise test; Echo, echocardiography; Exercise Echo, exercise echocardiography; NYHA, New York Heart Association.

Patient screening for trial eligibility will be based on reviews of medical records and echocardiographic results before baseline visits. After confirming study eligibility, baseline examinations (see the next section) will be conducted. Subsequently, patients will be randomized in a double-blind fashion to receive either udenafil or placebo (1:1) on top of any background therapeutic regimen in compliance with current recommendations [16], utilizing the randomization scheme depicted in Figure 2. An independent computer-generated list of random number will be used for participant allocations. Patients will receive either 50 mg udenafil or placebo twice daily for the first 4 weeks, and then if tolerated the dosage will be doubled to 100 mg twice daily for the next 8 weeks. Placebo and active study drugs (that is, udenafil) will appear to be identical and will be provided in blinded kits.

In the phase I trial, 42 healthy male volunteers received 25 to 300 mg of udenafil per day and experienced only minor adverse events [17]. Furthermore, a clinical trial on erectile dysfunction showed that 100 to 200 mg udenafil was well tolerated [18]. Because the half-life of udenafil is 7 to 9 hours, which is longer than that of sildenafil (4 hours), the study drug will be provided twice daily. Throughout the trial, medications prescribed by referring physicians will not be changed.

The ULTIMATE-SHF trial has been approved by the Clinical Research Institute at our hospital (H-1102-063-352), and the study drug, udenafil, has been approved as Investigational New Drug for therapeutic use in patients with chronic SHF, by the Korean Food and Drug Association.

### Blinding

Study patients, interviewers responsible for administering questionnaires, inspectors that conducted echocardiography and cardiopulmonary exercise tests (CPETs), and the study investigators and directors will be blinded from details of the randomization. Unblinding will be only accepted by the independent data and safety monitoring committee when a serious adverse event occurs, upon request from regulatory authorities, or when information regarding the allocation procedure is deemed important from the perspective of patient safety.

### Follow-up protocol

The trial duration is expected to be 3 months. Participants will attend study visits at screening, baseline, and weeks 4 and 12. At each visit, patients will undergo a complete physical examination, medical history-taking, and an assessment of drug compliance. Investigators will evaluate all clinical and laboratory adverse events at each visit. New York Heart Association functional class and predefined clinical events as described below will be recorded at each clinical visit. Venous blood will be drawn after a 15-minute rest period at screening, and at 4-week and 12-week visits, and will be subjected to the following test battery: B-type natriuretic peptide, complete blood count, electrolyte panel, serum creatinine, and liver function test. At screening visits, echocardiography will be performed at rest, and based on the results obtained study investigators will decide on patient eligibility. At baseline and at 12 weeks, participants will undergo CPET (V_Max_ 229 Respiratory Analyzer; SensorMedics, Homestead, FL, USA) and exercise echocardiography to evaluate exercise capacity in a quantitative manner. At all study visits, researchers will collect health-related information, such as adverse events, admission for acute decompensated HF, and change in medication. In addition, medication compliance of all participants will be carefully assessed by the pill-count method at every visit.

### Exercise test protocols

CPET and combined exercise echocardiography will be performed on a stationary cycle ergometer, since echocardiography should be performed at peak exercise [19]. Exercise echocardiography will be carried out after an overnight fast using a supine bicycle attached to a table tilted 30 to 45° to the left for best image acquisitions. Subjects will begin exercising at 25 W, and this will be increased by 25 W after 3 minutes. Workload in metabolic equivalent tasks will be calculated using a previously described standard equation for ergometer exercise [20]. Echocardiographic imaging will be performed continuously during each stage of the exercise protocol using a commercially available ultrasound system (GE vivid 7GE healthcare; Milwaukee WI, USA), and patients will be encouraged to exercise to maximum efforts. Breath-to-breath respiratory gas exchange will be continuously measured with a metabolic cart interfaced to the ergometer. Minute ventilation (V_E_, l/minute), carbon dioxide production (V_CO2_, ml/kg/minute), oxygen uptake (V_O2_, ml/kg/minute) and respiratory exchange ratio will be calculated. Peak V_O2_ is usually defined as the highest V_O2_ measured during the last minute of symptom-limiting exercise. The ventilatory anaerobic threshold will be determined using the V-slope method [21], and ventilatory efficiency will be determined by calculating the slope of the increase in ventilation with respect to carbon dioxide output (V_E_/V_CO2_), with values measured between at rest and the level of anaerobic threshold. To assess hemodynamic changes, echocardiography will be performed at rest and every 3 minutes during exercise by an expert echocardiographer with extensive experience of exercise echocardiography. LV dimensions, LV volumes, LV ejection fractions, mitral E and A velocities and corresponding E/A ratios, mitral annular E′, A′, and S′ velocities, E/E′ ratio, and pulmonary artery systolic pressure (obtained from maximal tricuspid regurgitation velocity determined using continuous wave Doppler) will be carefully obtained.

### Safety

Any participants with a hypersensitivity reaction to the study drug or an adverse event after study medication, those that do not want to remain in the trial, or those that comply with medication <80% will be dropped from the trial, as will those judged to be at risk due to participation in the trial by an attending physician.

Udenafil has been reported to be safe and well tolerated by healthy volunteers [17], and by patients with erectile dysfunction [14]. No serious adverse events occurred in either study. Common adverse events included nausea, headache, facial flushing, febrile sensation, penile erection, and eyeball pain, all of which have been reported to adverse events of other PDE5 inhibitors. Adverse events will be immediately reported directly to the study investigators, and study staffs will be obligated to manage these events. Patients will be interviewed at each visit about the occurrence of any adverse events, and onset time, duration, and severity will be recorded on case report forms. The possibility of causal relationships between the study drug and the intensities of adverse events will be evaluated by the investigators. The investigators are mandated to report serious adverse events to the institutional review board within 24 hours of recognition. A data and safety monitoring committee will monitor the safety of patients participating in the trial and all ongoing serious adverse events will be closely followed until these conditions have stabilized.

### Study withdrawals

Patients will be permitted to request withdrawal from treatment at any time without providing reasons. The primary investigator and the attending physician will also have authority to drop patients from the trial treatment if it is considered that further participation in the trial would be detrimental to the patient's well-being. Such treatment withdrawals will be documented using a case report form and in patient's medical records. In addition, we have a plan to evaluate the results of this trial in the middle of this study, and based on these interim results the trial can be prematurely terminated if the drug effect would be highly suggested to be beneficial or harmful.

### Study endpoints

The primary outcome will be changes in maximal V_O2_ measured at baseline and at 12-week visits. Maximal V_O2_ will be defined as highest oxygen uptake (averaged over five consecutive breaths) during the last minute of symptom-limited CPET. Secondary endpoints are detailed in Table 2.

**Table 2:** Secondary outcomes

	**Secondary outcomes**
1.	Changes of ventilator efficiency (V_E_/V_CO2_ slope)
2.	Ejection fraction
3.	E velocity of mitral inflow, E′ velocity, systolic mitral annular velocity (S′ velocity)
4.	E/E′ ratio
5.	Deceleration time
6.	Pulmonary artery systolic pressure measured by echocardiography at baseline and 12 weeks
7.	Post-exercise pulmonary artery systolic pressure measured by echocardiography at baseline and 12 weeks
8.	Symptomatic status (NYHA functional class and Borg dyspnea index)
9.	Plasma concentration of BNP assessed at baseline, 4 weeks, and 12 weeks
10.	Clinical endpoints, all-cause death, cardiac death, admission for heart failure, and the composites of these events will be assessed until the end of the study period
11.	Development of facial flushing, febrile sensation, eyeball pain, visual disturbance, headache, penile erection, intolerance or development of other adverse drug reactions related with study drug will be assessed

*BNP*, B-type natriuretic peptide; *NYHA*, New York Heart Association; V_E_, minute ventilation (l/minute); V_CO2_, carbon dioxide production (ml/kg/minute).

### Sample size calculation

Several studies have investigated the therapeutic efficacies of PDE5 inhibitors (the majority employed sildenafil as a study drug) in patients with chronic SHF [10,22]. Most clinical trials have adopted maximal V_O2_ to calculate the sample size and statistical power. In a study of 34 patients with SHF and pulmonary hypertension, Lewis and colleagues reported a 14% increase in maximal V_O2_ by sildenafil administration (from 12.2 ± 0.7 to 13.9 ± 1.0 ml/g/minute) [10]. On the other hand, in a study investigating the efficacy of sildenafil in 23 patients with SHF, Guazzi and colleagues reported an increase in maximal V_O2_ of approximately 21% (from 12.9 ± 6.8 to 15.6 ± 6.0 ml/minute/kg) [22].

Assuming a mean baseline maximal V_O2_ of 14.0 ml/kg/minute, a percentage increase in maximal V_O2_ of 0% in the placebo group and 15% in the udenafil group (data taken from the study by Lewis and colleagues [10] and our previous experiences), we anticipate final maximal V_O2_ values of 14.0 ml/kg/minute in the placebo group and of 16.1 ml/kg/minute in the udenafil group, with a conservative standard deviation for mean maximal V_O2_ of 2.5 ml/kg/minute. Accordingly, we calculated the sample size assuming a standard deviation of 2.5 ml/kg/minute using the following formula:

$$n_{i}=\frac{2{\left(Z_{a/2}+Z_{\beta }\right)}^{2}\sigma^{2}}{{\left(\mu_{1}-\mu_{2}\right)}^{2}}$$

Based on these assumptions and calculations, detection of the 15% increase in maximal V_O2_ by udenafil, with a power of 80% and a statistical significance level of 5%, will require 23 patients per group for a standard deviation of 2.5 ml/kg/minute. Thus, allowing for a loss of 10% (data are missing completely at random, which implies that the missing data are unrelated to the study variables), a maximum of 52 patients would be required or 26 patients per group.

### Statistical considerations

The principal analysis will be conducted on an intention-to-treat basis. We will analyze all patients according to the randomization scheme. However, data that are missing completely at random will be omitted without analysis. The differences between the treatment groups in the main outcome will be assessed using an unpaired *t* test adjusted by baseline values (analysis of covariance). Binary endpoints will be compared using Fisher’s exact probability test. For survival analysis and clinical events, Kaplan–Meier survival curves will be plotted and compared using the log-rank test based on a proportional hazards model using the chi-square test. Statistical significance will be accepted for *P* <0.05.

## Discussion

Vasoconstriction is a pathophysiological hallmark of chronic HF, which involves the systemic and pulmonary circulations, and results in increased impedance of the left and the right ventricular ejection. Defective nitric oxide release is a major contributor to vasoconstriction in chronic HF [6]. The potentiation of nitric oxide signaling thus represents an interesting pharmacological approach. Furthermore, the inhibition of PDE5, the predominant isoenzyme that metabolizes cGMP [23], has attracted interest as a potential therapeutic tool in chronic SHF. Experiences accumulated in patients with pulmonary arterial hypertension provided the rationale for a therapeutic PDE5 inhibition in chronic SHF. Since the first description of the favorable effects of PDE5 inhibitors in pulmonary arterial hypertension [7], the positive effects of PDE5 inhibition on cardiac remodeling and secondary pulmonary hypertension have raised the possibility that chronic PDE5 inhibition offers a potential adjunct to the current pharmacological management of chronic SHF [10,22,24]. In addition, cGMP upregulation by PDE5 inhibition has also been reported to modulate the contractility of hypertrophied right ventricles [25].

Sildenafil was the first PDE5 inhibitor introduced to the clinical arena, and thus has been extensively investigated in experimental and clinical cardiology research sectors. Bocchi and colleagues evaluated the efficacy of sildenafil for the treatment of erectile dysfunction in patients with chronic SHF, and found that sildenafil improved exercise capacity [26]. Furthermore, in a study of 13 chronic SHF patients of New York Heart Association class III, Lewis and colleagues showed that sildenafil improves maximal V_O2_ and reduces V_E_/V_CO2_ slope [10]. As a result of these studies, PDE5 inhibitors are now considered good candidates for the treatment of chronic SHF.

Some PDE5 inhibitor brands can now be prescribed. However, although these PDE5 inhibitors are similar in terms of mode of action and molecular structures, their potencies, pulmonary vessel selectivities, and half-lives differ [27]. As a representative example, the onset of action of sildenafil is rapid, and as mentioned above its plasma half-life is 4 hours [28,29], which means that patients take the medication three times a day. In contrast, the plasma half-life of udenafil is 7 to 9 hours, and thus medications can be reduced to twice daily so patient compliance should improve [30] and eventually prognosis should benefit [31]. In this trial, the relatively short-term effects of udenafil on the exercise capacity of chronic SHF patients will be evaluated. Notwithstanding, we believe that this study will open a door to new therapeutic prospects for the long-acting PDE5 inhibitor, udenafil, and will help provide a new therapeutic option for chronic SHF patients.

The proposed ULTIMATE-SHF trial, as outlined above, will be a randomized, placebo-controlled, double-blind clinical trial designed to investigate the therapeutic effect of a new, long-acting PDE5 inhibitor, udenafil, in patients with chronic SHF. If an improvement in exercise capacity, as determined by peak V_O2_, is demonstrated, udenafil could become a valuable therapeutic auxiliary for patients with chronic SHF that receive maximal medical management for SHF based on the current guidelines [16].

## Trial status

Patient recruitment.

## Abbreviations

cGMP: Cyclic guanine monophosphate; CPET: Cardiopulmonary exercise test; HF: Heart failure; LV: Left ventricular; PDE5: Phosphodiesterase type 5; SHF: Systolic heart failure; VE: Minute ventilation (l/minute); VCO2: Carbon dioxide production (ml/kg/minute); VO2: Oxygen uptake (ml/kg/minute)

## Competing interests

The authors declare that they have no competing interests.

## Authors’ contributions

KHK, HKK and ICH contributed to the study design and performed the analysis. HJC,HJK, HKK, SPL, YJK, and DWS recruited patients and interpreted data. KHK and HKK wrote the manuscript. All authors read and approved the final manuscript.
